# Cell electrofusion based on nanosecond/microsecond pulsed electric fields

**DOI:** 10.1371/journal.pone.0197167

**Published:** 2018-05-24

**Authors:** Chengxiang Li, Qiang Ke, Chenguo Yao, Yan Mi, Hongmei Liu, Yanpeng Lv, Cheng Yao

**Affiliations:** The State Key Laboratory of Power Transmission Equipment & System Security and New Technology, School of Electrical Engineering, Chongqing University, Chongqing, China; Universitat Zurich, SWITZERLAND

## Abstract

Traditionally, microsecond pulsed electric field was widely used in cell electrofusion technology. However, it was difficult to fuse the cells with different sizes. Because the effect of electroporation based on microsecond pulses was greatly influenced by cell sizes. It had been reported that the differences between cell sizes can be ignored when cells were exposed to nanosecond pulses. However, pores induced by those short nanosecond pulses tended to be very small (0.9 nm) and the pores were more easy to recover. In this work, a finite element method was used to simulate the distribution, radius and density of the pores. The innovative idea of “cell electrofusion based on nanosecond/microsecond pulses” was proposed in order to combine the advantages of nanosecond pulses and microsecond pulses. The model consisted of two contact cells with different sizes. Three kinds of pulsed electric fields were made up of two 100-ns, 10-kV/cm pulses; two 10-μs, 1-kV/cm pulses; and a sequence of a 100-ns, 10-kV/cm pulse, followed by a 10-μs, 1-kV/cm pulse. Some obvious advantageous can be found when nanosecond/microsecond pulses were considered. The pore radius was large enough (70nm) and density was high (5×10^13^m^-2^) in the cell junction area. Moreover, pores in the non-contact area of the cell membrane were small (1–10 nm) and sparse (10^9^-10^12^m^-2^). Areas where the transmembrane voltage was higher than 1V were only concentrated in the cell junction. The transmembrane voltage of other areas were at most 0.6V when we tested the rest of the cell membrane. Cell fusion efficiency can be improved remarkably because electroporation was concentrated in the cell contact area.

## Introduction

Cell fusion was defined as the process of combining two or more cells to form a combined cell. This process can occur naturally or be induced through biological, physical, or chemical means [1–5]. Cell fusion was a core technology of biological preparation (such as monoclonal antibody production)——Immune responses were induced in mice, after mice were injected with specific antigen proteins. The murine myeloma cells were fused with B lymphocytes and screened by a specific selection medium. On this medium, the unfused cells and the fusion of homologous cells will die. Only the fused hybrid cells can grow up normally. Finally, hybridoma cells were cultured in vitro or injected into the abdominal cavity of mice, so that a large number of monoclonal antibodies could be extracted from cell culture medium or mouse ascites.

According to the basic theory of electroporation, the transmembrane voltage (TMV) can be expressed as $U_{m}=\Delta \Psi =1.5aE_{0}cos\theta (1-e^{-t/\tau })\;$where *a* was the cell radius, *E*_*0*_ was the electric field, and *θ* was the angle between the electric field direction and the specified point. According to the formula of transmembrane potential, the TMV was positively correlated with the cell radius [6]. Under the same electric field condition, the TMV of large cells was higher than that of small ones. In other words, with the increasing of the electric field, the large cells will be electroporated prior to small cells [7–11].

Many simulations and experimental studies had shown that large numbers of nanoscale electroporation can be created on the cell membrane by using nanosecond pulses [12–20]. Electroporation degree was not affected by cell size when nanosecond pulses were used [21]. However, under the condition of nanosecond pulses, the pore sizes were small (1~10nm). Electroporation will recover rapidly before cell fusion occurred, owing to the small size of pores [22–26].

**Fig 1:**
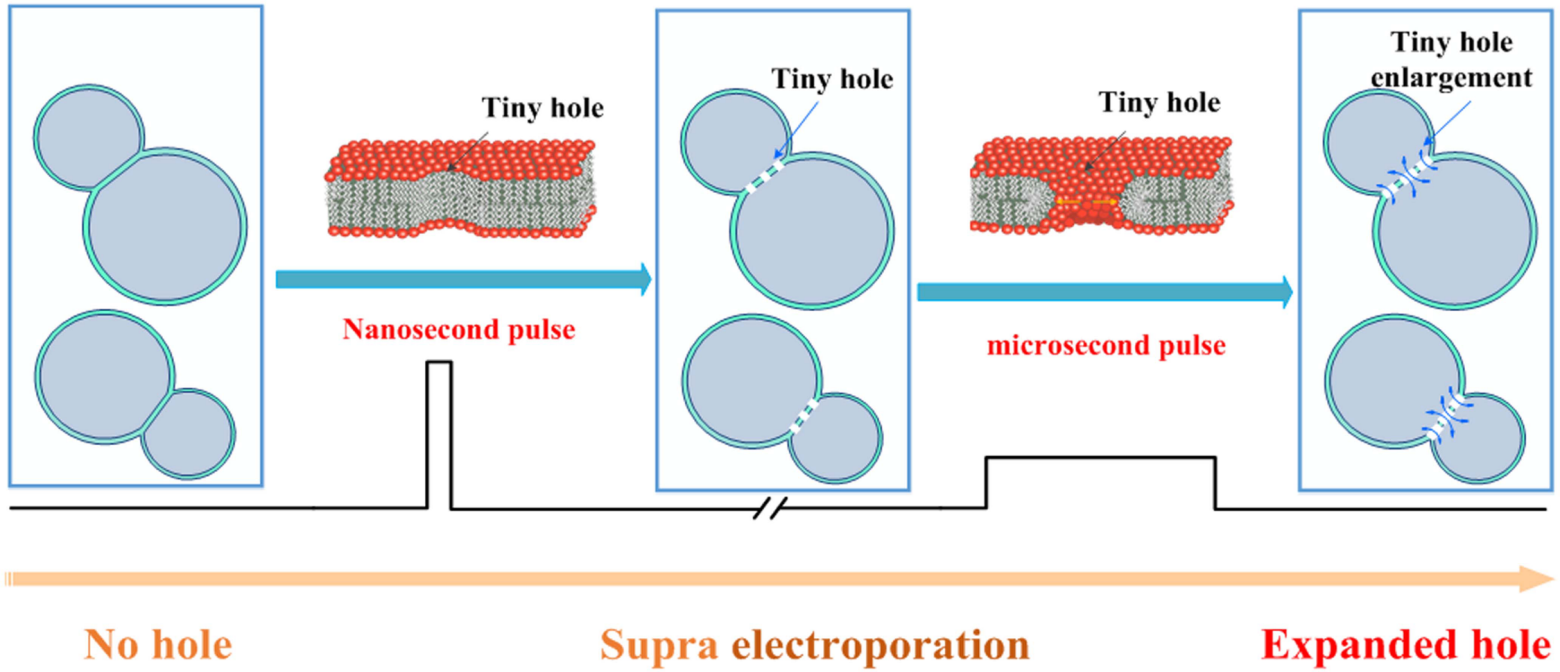
Schematic diagram of cell fusion using a sequential nanosecond/microsecond electric field pulse combination. 100-ns-long strong field pulse induced many tiny pores in the cell membrane, particularly in the junction region. After a brief delay, fusion process was followed by a low-field 10-microsecond pulse, which enlarged the pores.

In this paper, a novel view of “cell electrofusion based on nano/microsecond-pulse” was studied through simulation. This method combined the advantages of nanosecond pulse about cell size insensitivity and the ability of microsecond pulse concerning pores expansion and maintenance. Small pores of 0.7-1nm were created in the contact area of the cell membrane by using nanosecond pulse (100 ns). Then the μs pulse (10 μs) was applied to increase the size of the small pores to 50–70 nm and maintain the opening time of the pores. The schematic diagram of the nano/microsecond pulsed electrofusion was shown in Fig 1.

## Methods

To represent cell fusion in the production of monoclonal antibodies, cells of different sizes were simulated.

**Fig 2:**
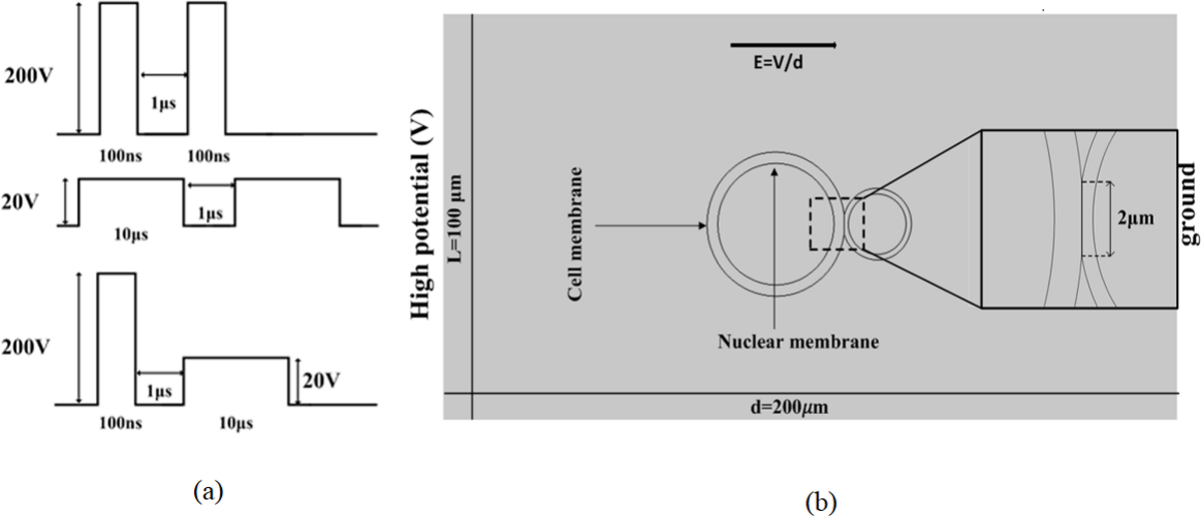
(a) Modeled electrical pulse shapes, magnitudes, and pulse width. (b) Geometry of the simulation. The two cells were contacted to each other in a rectangular 200-μm-long by 100-μm-wide frame. The inset was magnifying part of the cell junction area.

Two different sizes cells contacted with each other was established in COMSOL 5.2a software. The contact region was perpendicular to the electric field lines. The cell model was placed in a 200-μm-long, 100-μm-wide rectangle. The left boundary was high potential and the right boundary was ground. As illustrated in Fig 2B, length of the contact area was set to 2 μm (two dimensional model). In the figure, the large cell represented a myeloma cell with a 7.75-μm cell radius and a 6.54-μm nuclear radius. The small cell was the B lymphocyte with a 3.35-μm cell radius and a 3.25-μm nuclear radius. The extracellular region represented cell culture medium. The Electric Currents Interface of COMSOL was used to solve the transient currents and field distribution in the model domain of Fig 2B. The left boundary potentials shown in Fig 2A were input voltage. The average field strengths were 10 kV/cm for the two-100ns pulses, and 1 kV/cm for the two-10μs pulses. For the combined pulses, fields were 10 kV/cm for 100 ns followed by 1 kV/cm-10μs. The formula with equal dose was used in this paper. $\text{Dose}=\Sigma_{n=1}^{N}V_{n}^{2}\times \text{Tn}\left[V^{2}\text{s}\right]$.[27], where *V*_*n*_ was the voltage of the *n*th pulse, *T*_*n*_ was the duration of the *n*th pulse, and *N* was the total number of pulses. Fig 2A showed the pulse waveform diagram.

200V×200V×100ns×2 = 20V×20V×10μs×2 = 200V×200V×100ns×1+20V×20V×10μs×1 = 8000[V^2^μs]

The electric field distribution was set up throughout the model region. To determine the field distribution inside the cells, electrodynamical equations of the cells must be solved. Assuming that the potential at any point on the spherical cell membrane was ψ, according to the electromagnetic field theory, the potential inside and outside the cell membrane obeyed the formula.

$$-\nabla \left(\sigma_{i}\nabla \Psi \right)-\varepsilon_{0}\varepsilon_{r}\nabla \left(\frac{\partial \left(\nabla \Psi \right)}{\partial t}\right)=0$$ (1)

In Eq (1), *σ*_*i*_ represented the conductivity of the given location (including intracellular media, membrane, and external media), *ε*_*0*_ was the permittivity of the vacuum, *ε*_*r*_ was the relative dielectric constant, *t* was time, and ∇ was the spatial gradient operator. The Δ*ψ* (TMV) was the difference between the membrane’s internal and external voltage

$$\Delta \Psi =\Psi_{i}\left(t\right)-\Psi_{0}\left(t\right)$$ (2)

The subscript *i* stood for internal and *o* stood for external. Under the action of the electric field, electroporation of a membrane can be described as the formation of hydrophilic micropores in lipid bilayers. Under certain electric field conditions, with the pore density increasing, membrane conductivity and permeability would be increased. The formation of the pores provided new channels for transmembrane current. The transmembrane current density can be expressed by *J*_*EP*._ The overall transmembrane current density can be expressed as

$$J\left(t\right)=\frac{\sigma_{m0}(\Delta \Psi )}{d_{m}}+\frac{\varepsilon_{0}\varepsilon_{m}}{d_{m}}\frac{\partial (\Delta \Psi )}{\partial t}+J_{EP}\left(t\right)$$ (3)

where *σ*_*m0*_ was the conductivity of an unelectroporated membrane and *d*_*m*_ was the membrane thickness. De Bruin and Krassowska [28] proposed the formula for *J*_*EP*_:

$$J_{EP}\left(t\right)=i_{EP}\left(t\right)N\left(t\right)$$ (4)

$$i_{EP}\left(t\right)=\Delta \Psi \sigma_{p}\pi r_{p}^{2}\frac{A}{d}$$ (5)

$$A=\frac{e^{v_{m}}-1}{\frac{e^{v_{m}}\left(w_{0}e^{w_{0}-nv_{m}}-nv_{m}\right)}{w_{0}-nv_{m}}-\frac{\left(w_{0}e^{w_{0}+nv_{m}}+nv_{m}\right)}{w_{0}+nv_{m}}}$$ (6)

*σ*_*p*_ was the conductivity of the solution inside the pores, *r*_*p*_ was the pore radius, *i*_*EP*_ was the current flowing through a single pore, *and N* was the pore density. $v_{m}=\Delta \Psi =(F/RT)$, where *F* was the Faraday constant, *R* was the gas constant, and *T* was the absolute temperature, all these parameters were shown in Table 1. The dynamic change of the pore density *N* was Smoluchowsky equation [29]:

$$\frac{dN(t)}{dt}=\alpha e^{{\left(\frac{\Delta \Psi \left(t\right)}{V_{ep}}\right)}^{2}}\left(1-\frac{N\left(t\right)}{N_{0}}e^{-q{\left(\frac{\Delta \Psi \left(t\right)}{V_{ep}}\right)}^{2}}\right)$$ (7)

*N(t)* indicated pore density on the membrane, *N*_0_ was the equilibrium pore density, and *α*, *q* and *V*_*ep*_ were constants (whose specific values were in Table 1). *V*_*ep*_ determined the TMV threshold $\Delta \Psi_{c}$. The relationship between *V*_*ep*_ and $\Delta \Psi_{c}$ was given in [30]. In most studies, $\Delta \Psi_{c}\;$was between 500 and 1000 mV. In this work, $\Delta \Psi_{c}$ = 1000 mV (*V*_*ep*_ = 258 mV) was selected.

**Table 1:** Model parameters.

Parameter	Symbol	Value	
**cell membrane thickness**	d_mem_	5nm	[28]
**Equilibrium pore density**	N_0_	1.5×10^9^/m^2^	[28]
**characteristic voltage of electroporation**	V_ep_	258mV	[28]
**energy barrier within pore**	w_0_	2.65	[28]
**relative entrance length of pore**	n	0.15	[28]
**Large cell radius**	r_c_	7.75μm	[31]
**Large nuclear radius**	r_n_	6.54μm	[31]
**Small cell radius**	r_c2_	3.85μm	[31]
**Small nuclear radius**	r_n2_	3.25μm	[31]
**Extracellular fluid conductivity**	$\sigma_{e}$	0.01S/m	[31]
**Faraday's constant**	F	9.65×10^4^ C/mol	[32]
**gas constant**	R	8314 J/Kmol	[32]
**absolute temperature**	T	295 K	[32]
**Cytoplasmic conductivity**	$\sigma_{c}$	0.25 S/m	[33]
**Cytoplasmic permittivity**	$\varepsilon_{c}$	70	[33]
**Nucleoplasmic conductivity**	$\sigma_{n}$	0.5 S/m	[34]
**Cell membrane conductivity**	$\sigma_{m0}$	5×10^−7^ S/m	[35]
**Cell membrane permittivity**	$\varepsilon_{mem}$	4.5	[35]
**Nuclear membrane conductivity**	$\sigma_{ne}$	1×10^−4^ S/m	[36]
**Extracellular medium permittivity**	$\varepsilon_{m}$	80	[37]
**Nuclear membrane permittivity**	$\varepsilon_{ne}$	7	[38]
**minimum radius of hydrophilic pores**	rr	0.51nm	[39]
**Nucleoplasmic permittivity**	$\varepsilon_{np}$	70	Set equal to $\varepsilon_{c}$

Put Eq (5), (6), (7) into Eq (4), get (8)

$$J_{EP}\left(t\right)=\frac{\left(\Psi_{i}-\Psi_{0}\right)}{d_{m}}\sigma_{m}\left(t\right)+\frac{\varepsilon_{0}\varepsilon_{m}}{d_{m}}\frac{\partial (\Psi_{i}-\Psi_{0})}{\partial t}$$ (8)

The size of the membrane conductivity *σ*_*m*_ was related to the degree of pores. As the membrane was electroporated, both its permeability and conductivity would change. The conductivity was

$$\sigma_{m}\left(t\right)=\sigma_{m_{0}}+N\left(t\right)\sigma_{p}\pi r_{p}^{2}A$$ (9)

From Eq (3), *σ*_*m0*_ represented the initial value of conductivity (5×10^−7^ S/m). Therefore, the total conductivity *σ*_*m*_ was obtained by the sum of its dynamic conductivities. The above formula reflected the relationship between the dynamic change of the membrane conductivity and the TMV and pore density. *N(t)* was the pore density, *σ*_*p*_ was the conductivity of the solution inside the pore, and *r*_*p*_ was pore radius. The pore radius dynamics [30] were given by

$$\frac{dr_{j}}{dt}=\frac{D}{kT}\left(\frac{\Delta \Psi^{2}F_{max}}{1+r_{h}/\left(r+r_{t}\right)}+4\beta {\left(\frac{r_{*}}{r}\right)}^{4}\frac{1}{r}-2\pi \gamma +2\pi \sigma_{eff}r\right)$$ (10)

It had been reported that the lower the cell culture medium conductivity was, the better the fusion effect would be. Some reports had changed the extracellular fluid conductivity to 0.001 S/m to control the effect of cell fusion [40]. In practice, there was a non-ignorable flaw in this method. When the extracellular solution conductivity was too small, the volume of cell would change, likely resulting in loss of cell vitality even cell death. Therefore, conductivity of 0.01S/m was selected in our simulation, which was commonly used in cell fusion experiments. The efficacy of electroporation on cell fusion was judged by two criteria. First, larger size pores should be concentrated at the cell junction, with few or none pores in other areas of the membrane. Second, the pore density should be high enough at the cell junction, and low elsewhere.

Based on the above two standards, the cell membrane pore radius, pore density, and TMV were simulated. The effects of the three different pulse forms described in Fig 2A were compared.

## Results

### I. Cell electroporation radius

**Fig 3:**
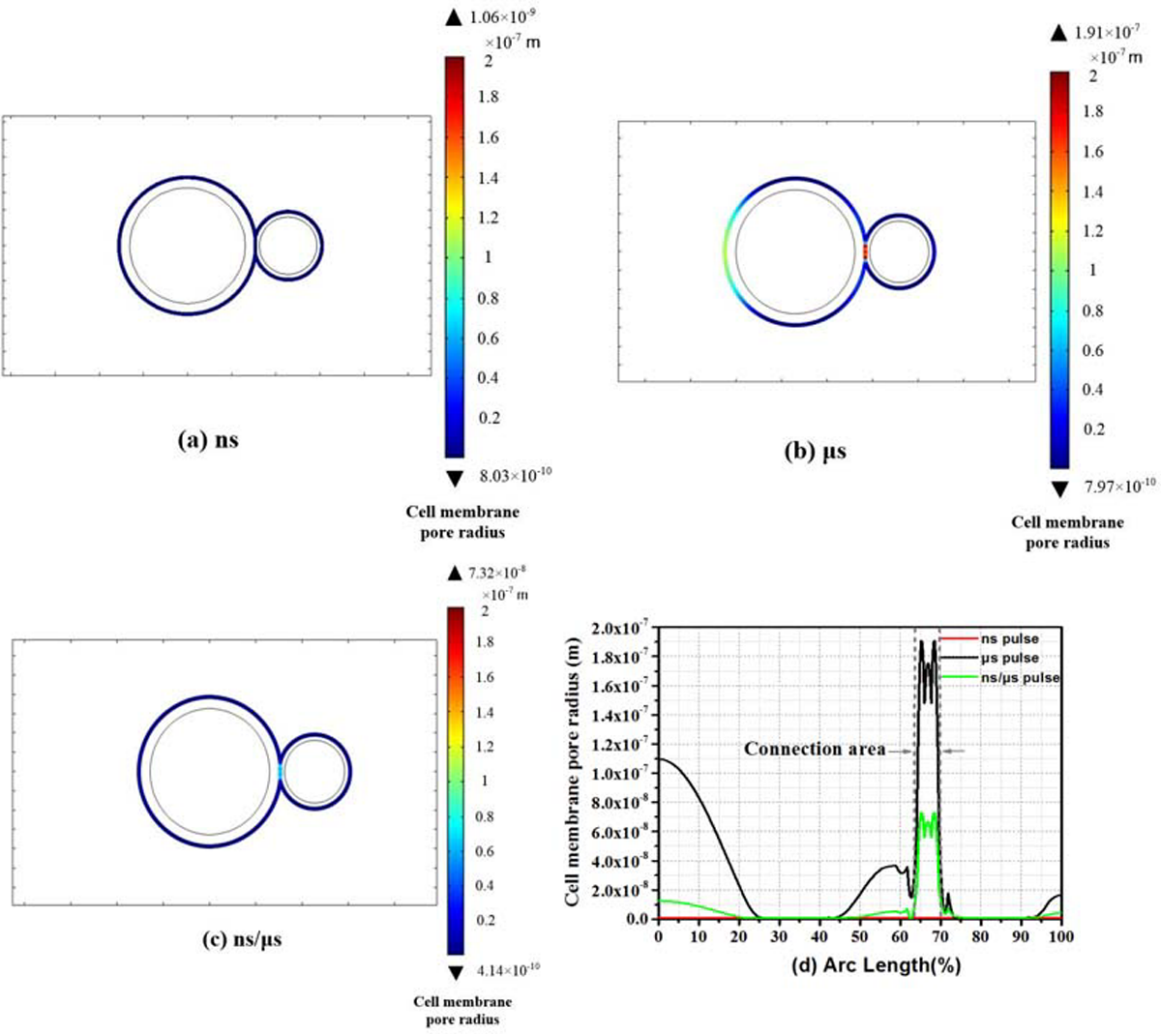
Distribution of pore radius along the two-cell membrane. (a) Results of the nanosecond pulses, (b) the microsecond pulses, and (c) the nanosecond/microsecond pulses. (d) Graphical overlay of the results of the three pulses.

The model with three different pulses were simulated respectively, according to the cell model in Fig 2B [31]. Each of the pulse shapes was showed in Fig 2A. The distributions of pore radius along the cell membranes were shown in Fig 3A–3C. The color bar of Fig 3A–3C indicated numerical value of pore radius. The pore size induced by nanosecond pulses in Fig 3A was much smaller than the other two results (Fig 3B and 3C).

**Fig 4:**
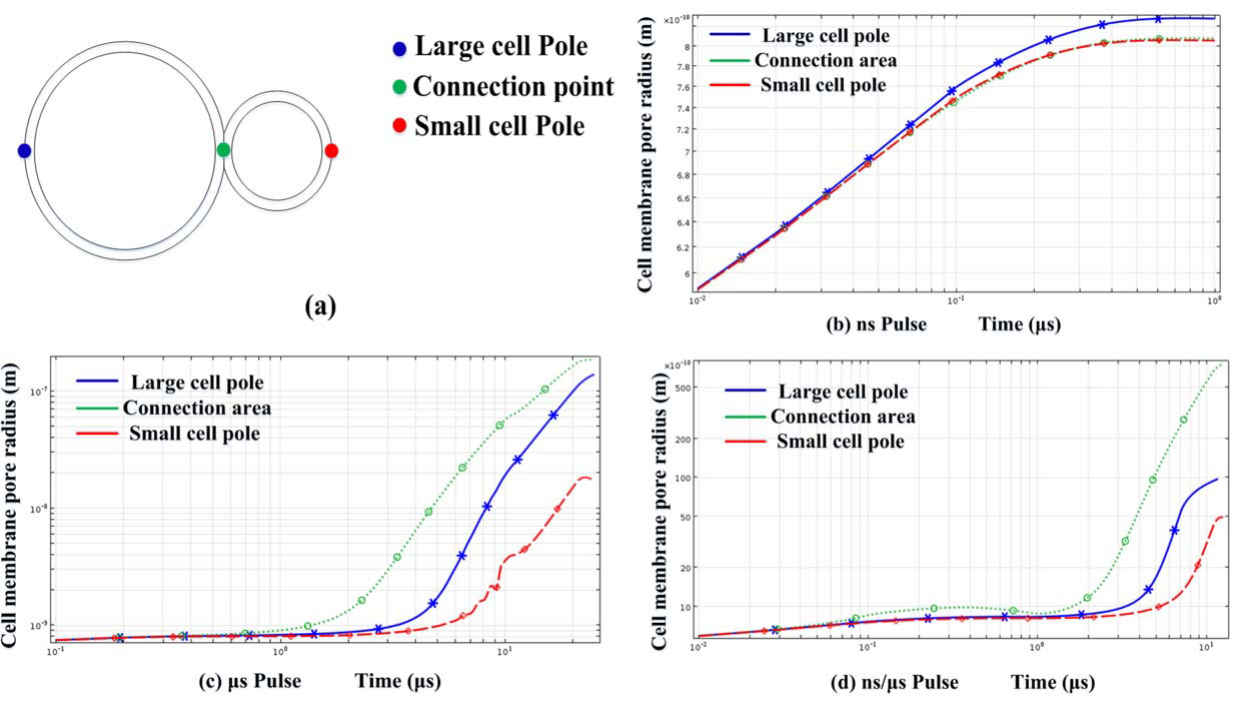
Time evolution of the pore radius at three locations selected along the two-cell membrane was shown. In (a). Blue represented the large cell pole, green represented the midpoint of the two-cell junction region, and red represented the small cell pole. (b) Results of the nanosecond pulses, (c) the microsecond pulses, and (d) the combined nanosecond/microsecond pulses.

From Figs 3A, 3D and 4B, the pore radii along the surface of the cell membrane were almost in the same level (0.9nm) by only applying the nanosecond pulses. This result substantiated the viewpoint that effects under nanosecond pulses were insensitive to cell size, which was of benefit to fusing the cells with different sizes. However, pores produced by nanosecond pulses were around 0.9 nm, and it was difficult for DNA and other macromolecules to pass through these channels.

The study [22] found that only nanometer-size pores could be created by nanosecond pulses, and the size of pores were small which were apt to recover easily. Identical conclusions can be obtained by using multi cell dielectric simulation based on Gowrishankar’s Transport Lattice Model [41–43].

By using microsecond pulses, distribution of the pore radius was showed in Figs 3B, 3D and 4C. The pores at cell junction area were large (nearly 180 nm), but large size pores had also been found in other parts of the cell membrane. 70–110 nm pores could be found near the poles of large cells and 45° point. Besides, pores at the large cell pole (100~110nm) were much larger than the small cell pole (10~20nm), which supported the point that the degree of electroporation was related to size of cells when applied microsecond pulses. Additionally, percentage of pores which were above 20-nm radius accounted for 50%. Such a severe electroporation rate of membrane would result in a high mortality before the cells were fused.

By using nanosecond/microsecond pulses, a large number of pores can be created, which can be enlarged during the longer μs pulse. The distributions of cell membrane pore radius were showed in Figs 3C, 3D and 4D. Large pores were mainly located in the junction areas. The pore radius was about 60-70nm, which was large enough to promote cell fusion. Outside the area, pore radius remained small (1-10nm), which can be regarded as no obvious electroporation.

In Fig 3D, the pore radii, along the cell membrane applied by three kinds of pulses, were compared. The cell junction area was labelled by a pair of dashed vertical lines. The red curve represented the result for the nanosecond pulses. Although pores were mainly concentrated in the contact area, the average value of pore radius was extremely small, around 0.9 nm. The black curve stood for the microsecond pulses. The green curve showed the result of the nanosecond/microsecond pulses. All the large pores (60~70nm) were concentrated in the cell junction area.

Time evolution of the pore radius was shown in Fig 4, and three locations were selected along the two-cell membrane.

### II. Analysis of pore density

**Fig 5:**
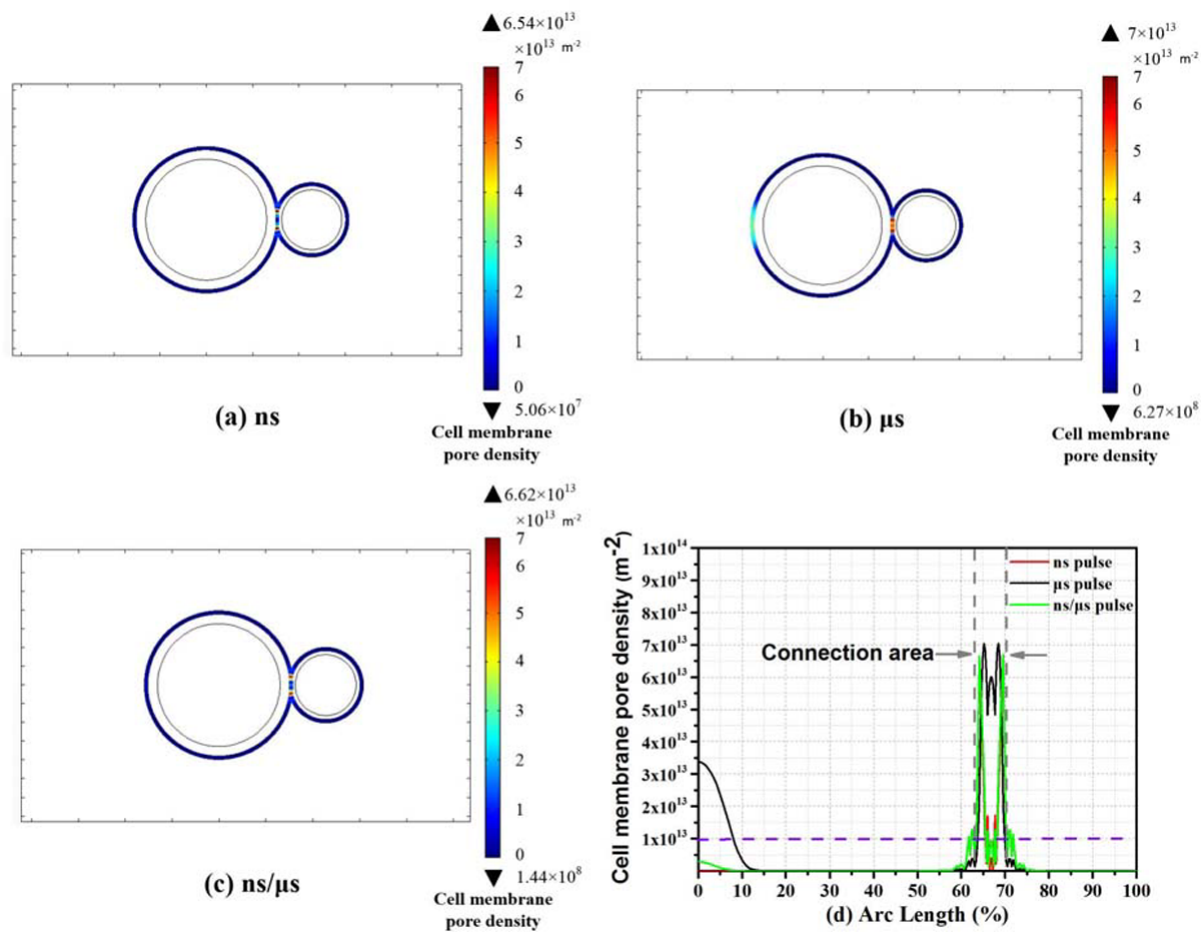
(a-c) Two-dimensional pore density distributions along the surface of the two cell membranes. (d) Graph of pore densities along the surface of the two cell membranes. The dashed gray lines indicate the cell contact area.

**Fig 6:**
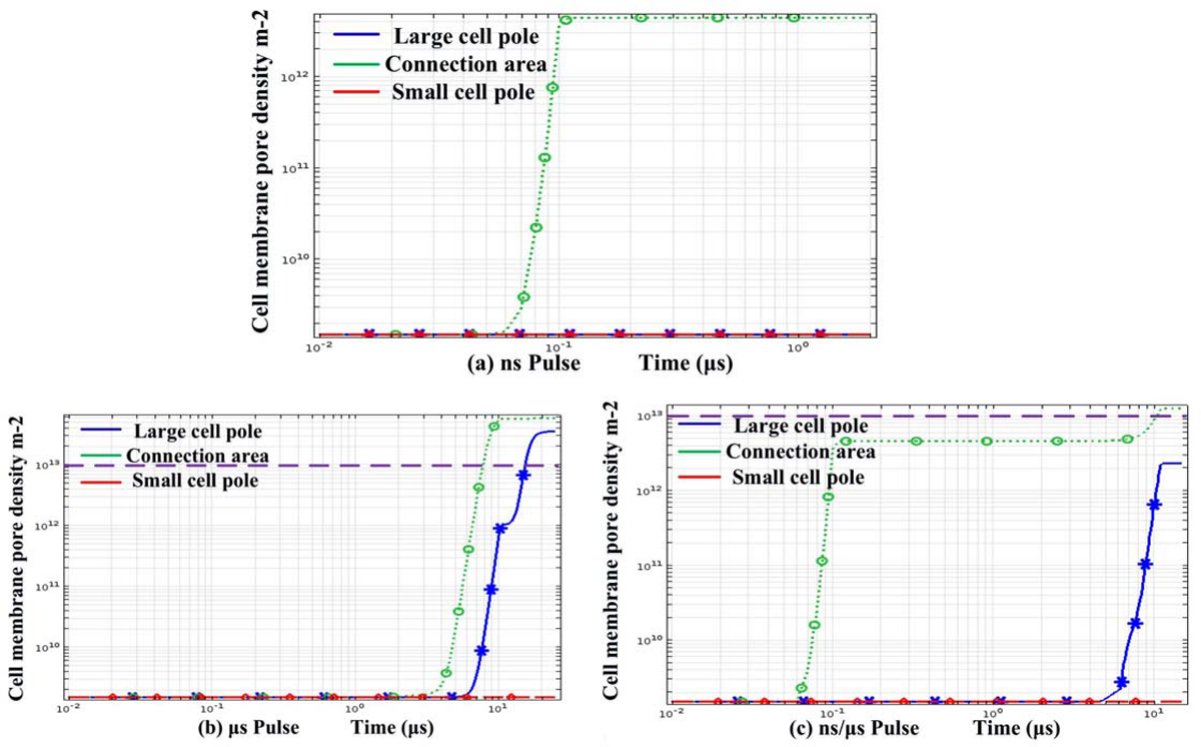
Nanosecond pulse results were shown in (a), the microsecond pulse in (b), and the pulse combination in (c). The dashed purple line represented a pore density of 10^13^ m^-2^.

It was not sufficient to evaluate the efficiency of cell electrofusion merely through the pore radius results. Small pore density may exist in the cell membrane accompanied with large radius pores. Alternatively, areas with small pore radius may possess of large density of pores. Both of these cases were not conducive to cell fusion. Therefore, the pore density was supposed to be studied. The distribution of pore density along the cell membranes was showed in Fig 5, and the time evolutions of pore density at the poles and junction center were expressed in Fig 6. Research Report [29,31,36,44] indicated that electroporation occurred when the pore density increased of four order of magnitude with respect to the initial value. 1.5×10^9^/m^2^ was chosen as the initial value of the pore density, so the value of pore density which was higher than 10^13^ m^-2^ can be regarded as pore density threshold of electroporation.

Results of nanosecond pulses were shown in Figs 5A, 5D and 6A. Pores were concentrated in the cell junctions, in where pore density was about 1×10^13^ m^-2^. However, the value nearly remained unchanged (10^9^m^-2^), when considering the region outside the two-cell contact area.

Pore density induced by microsecond pulses was given in Figs 5B, 5D and 6B. In the cell junction area, the pore density reached 6×10^13^m^-2^. Moreover, the value of the two cell poles was also extremely large, around 3×10^13^m^-2^. By using microsecond pulses, the trend that large area electroporation was created on cells was consistent with the experimental results of Damijan Miklavčič et al [31].

By using the nanosecond/microsecond pulses, Figs 5C, 5D and 6C showed that the density in the contact area can reach 5×10^13^ m^-2^. Other regions of the membrane perimeter such as the equator, poles, and 45° point, reached only 10^9^−10^12^ m^-2^, which can be considered as no obvious electroporation. As shown in Fig 6C, the pore density changing with time evolution had a two-step character, the first stage: after nsPEF, pore density rose rapidly. Before μsPEF stimulation, the pore density remained unchanged. The second stage: after μsPEF stimulation, pore density continued to rise.——Nanosecond pulse created pores and microsecond expanded the pores.

### III. Transmembrane voltage analysis of the cell membrane

**Fig 7:**
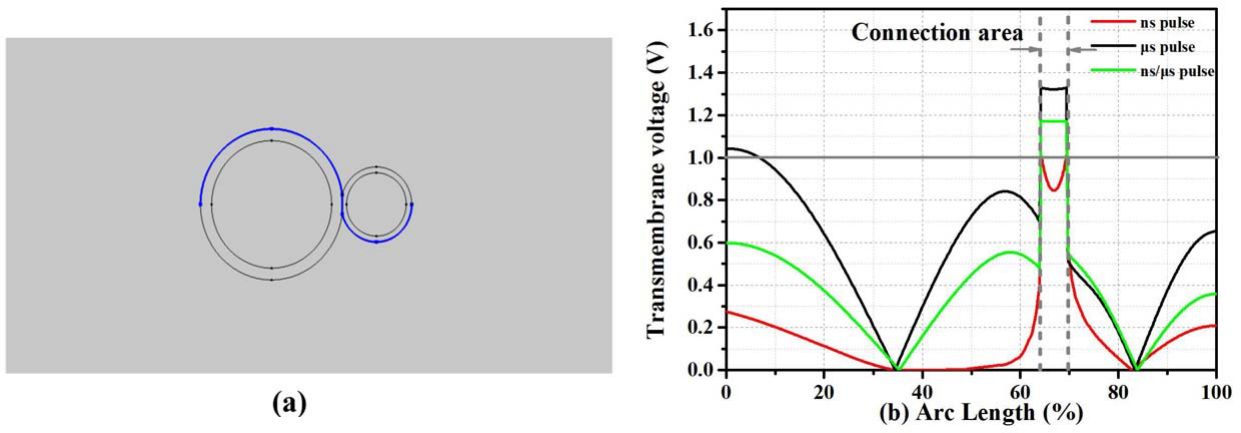
(a) represented the TMV simulation region. In 7(b), the red, black, and the green curves represented the TMV under the nanosecond pulse, the microsecond pulse, and the nanosecond/microsecond pulse combination respectively.

After the boundary of a phospholipid bilayer membrane was charged, the TMV will form on the surface of the cell membrane. When the TMV reached a certain threshold (typically around 1V), many nanometer sized pores would be created on the cell membrane. At this point, the conductivity of the cell membrane would suddenly increase by several orders of magnitude. With the pore density changing, the membrane conductivity would eventually affect the TMV. Fig 7A showed the simulation area of TMV along the surface of the cell membrane. In Fig 7B, the solid gray line represented the TMV threshold (1V), and the dotted gray lines denoted the cell junction area. It can be regarded as electroporation if the TMV exceeded 1V, otherwise it was considered as no obvious electroporation. Results of the microsecond pulse showed that TMV of both large cell pole and contact area were above 1V. However, when the nanosecond pulse was applied, the TMV can reach only about 0.85-1V in the junction. The TMV of the connected area was higher than the threshold 1V and it was lower than 0.6V elsewhere by using the nanosecond/microsecond pulses.

## Discussion

The purpose of this simulation was to propose and demonstrate a novel conjecture of cell electrofusion method based on composite pulses. Small pores in the cell junction area can be created by nanosecond pulses. Then the microsecond pulse was applied to enlarge the radius of pores which were located in the cell junction area. According to the literature [27, 45], high field intensity pulse (nanosecond pulses) were mainly contributed to increasing the number of pores, but less contributed to enlarging the pore radius. However, wide pulse width pulse (microsecond pulses) were mainly contributed to enlarging the pores, but less contributed to increasing the pore density. The nanosecond pulse took advantages of the cell-size-insensitivity, and microsecond pulse possessed of the superiority about enlargement of the pores. So we wanted to know whether we can combine nanosecond pulses with microsecond pulses and make use of the advantages of these two pulses. Therefore simulations were built to verify our conjecture. Some research reports could verify our simulation. Through molecular dynamics simulation, Hu et al. [12–14] found that the nanoscale micropores of cell membrane can be produced by using nsPEF of the field intensity 100kV/cm and the pulse width 10ns. By using field strength 40kV/cm, pulse width 10ns PEF, Silve et al. [15] found: pores can be created by nsPEF, but the size of the micropores was too small to allow the large molecules to pass. The dye molecule can not enter into the cell through the cell membrane as well. In other words, nsPEF can actually produce a large number of tiny pores on the surface of the cell membrane.

Damijan Miklavčič et al [31] had proven cell fusion could be realized by nsPEF. However, in his report, the fusion efficiency was low (8.4%). It was because the size of the pore on the surface of the cell membrane produced by nsPEF was too small (nanoscale micropores), so that the procedure of cell fusion was prevented.

Simulation results of Damijan Miklavčič et al [31] showed that the rate of cell fusion was in the low level because of obvious difference in cell size when applying μsPEF. Recently, Professor Richard Heller of Old Domonion University in the United States found [46]: After cells were simulated with 32kV/cm, pulse width 60ns, repetition rate 1Hz nsPEF and then simulated with 800V/cm, pulse width 5ms millisecond pulsed, and gene transfection efficiency can be significantly improved compare with efficiency under high voltage nsPEF electric field. However, the effect of improving efficiency can not be achieved if the order of the low voltage pulse electric field and high voltage pulse electric field was exchanged. Significant difference in gene transfection between ns/μs and μs/ns proved our point── nsPEF can create a large number of nanoscale pores on the cell membrane, and the pore radius of the cell membrane can be enlarged and maintained by μsPEF [47]. At the same time, by using pulsed electric field to exterminate bacteria, Žgalin et al [48] found sterilizing effect under field strength 80kV/cm, pulse width 10ns PEF was not good. However, sterilizing effect can be significantly increased, when combining ns PEF with μs PEF. According to the above study, we fully believed ns/μs can control the size of pores by proper selection of pulse parameters.

According to Damijan Miklavčič et al [31], range of nanosecond pulse electrofusion parameters: pulse length 100~200ns, number of pulses 1~20, electric field amplitude 5~10kV/cm. Pulse length 100ns, amplitude 10kV/cm was selected in this simulation. Parameters of microsecond electrofusion commonly used in the production of monoclonal antibodies: pulse length 10~40μs, number of pulses: 1~2, electric field amplitude: 1~3kV/cm. Pulse length 10μs, amplitude 1kV/cm was selected in this simulation, in order to save simulation time.

Electroporation was affected by cell size under microsecond pulse. Hence, as for different size cells fusion, when the same electric field was applied, the TMV of the large cell membrane was higher than the TMV of the small cell. In other words, the large cell may have died when the small cell was electroporated. This would lead to low efficiency when only using microsecond pulses to produce monoclonal antibodies.

**Fig 8:**
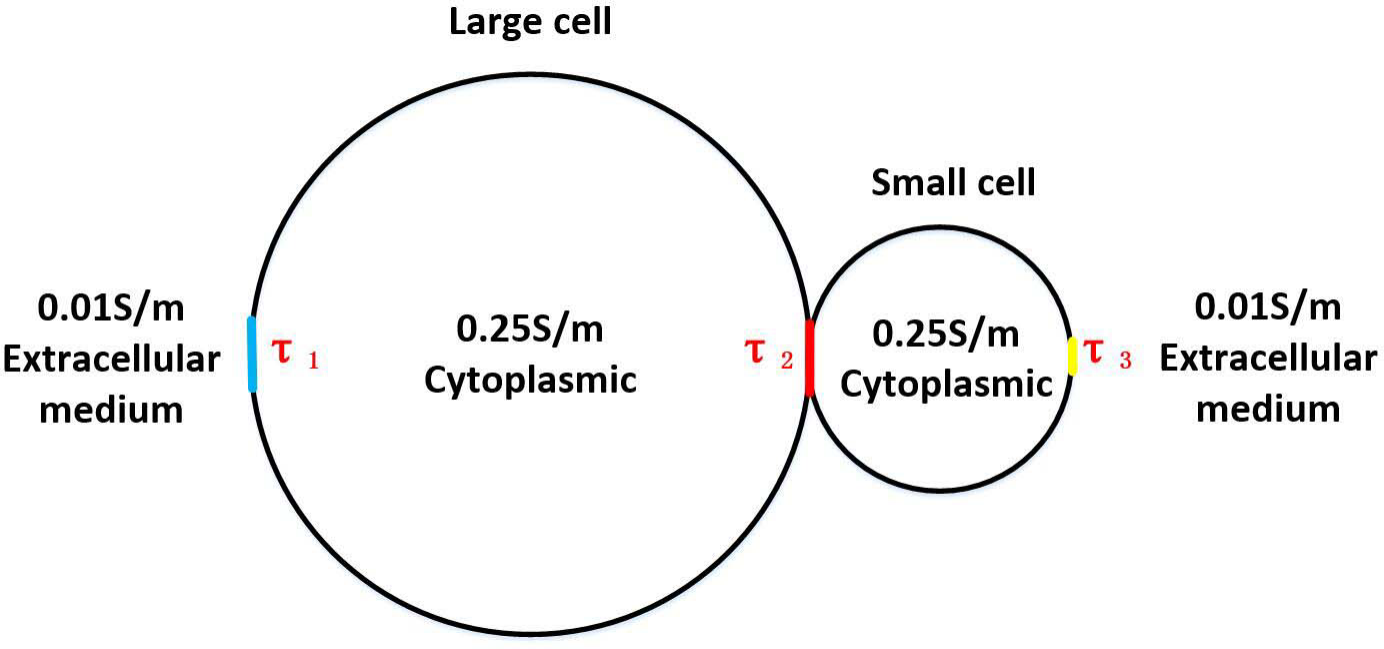
Calculation model of charge time constant.

The way by using 100-nanosecond pulses avoided this problem. The charging time constant of the cell membrane was $\;\tau =aC_{m}\left(1/\sigma_{c}+1/\sigma_{i}\right)$, where *a* was the cell radius, *C*_*m*_ was the membrane capacitance, and *σ*_*c*_ and *σ*_*i*_ were the conductivities of the intracellular fluid and extracellular fluids respectively. According to Fig 8, the charge time constant of the cell membrane can be calculated.

According to charging time constant of the cell membrane, we can figure out τ of pole of large cell, contact area and pole of small cell were 8.06μs, 0.46μs, 4.006μs respectively. The time constant of contact area was small (0.46μs far less than the time constant of cell poles), which explained why the number of pores in contact area was much higher than poles of cell by using nanosecond pulse. But if nanoscale pulse with a high field strength was used, there would cause another problem──The pores created by ns pulse were too small to fuse. Nanosecond pulses were mainly contributed to increasing the number of pores, however, microsecond pulses were mainly contributed to enlarging the pores. Therefore when using nanoscale/microsecond pulse, nanoscale pores on the cell membrane were created by nanoscale pulse, and then the pores could be enlarged by using low field strength microsecond pulse.

When the nanosecond/microsecond pulse was applied, the TMV was below the threshold of TMV (1V) along most of the cell perimeter, while the nanometer-scale pores were generated in the junction area. Studies had shown that using a lower voltage for sufficient time would increase electroporation efficiency [49]. With appropriately selecting field strengths and pulse delay to promote pore growth in the junction area, we can control the size of the pores in the contact area to enhance cell fusion efficiency.

In order to verify this conjecture, In the future, we would have a lot of experiments to verify our simulation.

## Conclusion

Based on the complementary advantages of nanosecond and microsecond pulses in electroporation, a novel idea of nanosecond/microsecond composite pulses to induce cell electrofusion was proposed. Numerical simulations results about cell fusion were showed in this work.

A large number of pores can be generated at the cell junctions (10^13^m^-2^) by using 100-nanosecond pulses, but the size of pores were too small (around 0.9nm). This was not conducive to cell fusion, because macromolecules such as DNA was difficult to pass through nanoscale micropores.

Many pores (6×10^13^ m^-2^) with large radius (180 nm) can be created in the cell contact area by using microsecond pulses. However, the pore density (3×10^13^ m^-2^) and pore radius (110 nm) were also extremely high when we simulated the non-contact area of two cells. This phenomenon may lead to excessive mortality by microsecond pulses.

Pores, pore density 5×10^13^ m^-2^ and pore radius 60–70 nm, were created in the contact area of the cells by using the nanosecond/microsecond pulses. What’s more, there was no obvious electroporation elsewhere along the two cell membranes. The nanosecond/microsecond composite pulses technique not only retained advantages of the nanosecond pulse in the cell-size-insensitivity, but also made use of the ability of microsecond pulses in expansion of pores. This method can greatly improve the efficiency of cell electrofusion, and provide an effective means to carry out cell fusion.
